# The m.9143T>C Variant: Recurrent Infections and Immunodeficiency as an Extension of the Phenotypic Spectrum in *MT-ATP6* Mutations?

**DOI:** 10.3390/diseases8020019

**Published:** 2020-06-09

**Authors:** Diana Lehmann Urban, Leila Motlagh Scholle, Matias Wagner, Albert C. Ludolph, Angela Rosenbohm

**Affiliations:** 1 Department of Neurology, Ulm University, 89081 Ulm, Germany; albert.ludolph@rku.de (A.C.L.); angela.rosenbohm@uni-ulm.de (A.R.); 2 Department of Neurology, University of Halle/S., 06120 Halle, Germany; leila.scholle@medizin.uni-halle.de; 3 Institute of Human Genetics, School of Medicine, Technical University Munich, 81675 Munich, Germany; Matias.Wagner@mri.tum.de; 4 Institute of Neurogenomics, Helmholtz Zentrum München, 85764 Munich, Germany

**Keywords:** *MT-ATP6*, mitochondrial disease, immune deficiency, phenotypic spectrum

## Abstract

Pathogenic variants in the *MT-ATP6* are a well-known cause for maternally inherited mitochondrial disorders associated with a wide range of clinical phenotypes. Here, we present a 31- year old female with insulin-dependent diabetes mellitus, recurrent lactic acidosis and ketoacidosis recurrent infections with suspected immunodeficiency with T cell lymphopenia and hypogammaglobulinemia as well as proximal tetraparesis with severe muscle and limb pain and rapid physical exhaustion. Muscle biopsy and respiratory chain activities were normal. Single-exome sequencing revealed a variant in the *MT-ATP6* gene: m.9143T>C. Analysis of further specimen of the index and mother (segregation studies) revealed the highest mutation load in muscle (99% level of mtDNA heteroplasmy) of the index patient. Interestingly, acute metabolic and physical decompensation during recurrent illness was documented to be a common clinical feature in patients with *MT-ATP6* variants. However, it was not mentioned as a key symptom. Thus, we suggest that the clinical spectrum might be expanded in *ATP6*-associated diseases.

## 1. Introduction

Pathogenic mitochondrial DNA (mtDNA) variants are associated with a wide range of clinical phenotypes, often involving multiple organ systems. Mitochondria are known to be the “powerhouse of the cell”, by providing cellular energy (adenosine triphosphate (ATP)). One indispensable component of this is complex V (CV, ATP-synthase), which is composed of 15 structural and two assembly subunits encoded by both the mitochondrial DNA (mtDNA; ATP6 and ATP8) and nuclear genome (15 subunits) [1]. The *MT-ATP6* gene encodes for a subunit of the key enzyme complex V [2].

Variants in *MT-ATP6* are a well-known cause for maternally inherited mitochondrial disorders associated with a continuous spectrum of clinical phenotypes [2,3], e.g., clinically grouped into Leigh syndrome (LS) [4,5], neuropathy, ataxia and retinitis pigmentosa syndrome (NARP) [6,7], Charcot–Marie–Tooth (CMT) disease-like pure peripheral neuropathy [8], and spinocerebellar ataxia (SCA) with upper motor neuron signs [9,10]. 

A recent cohort study reported novel findings associated with *MT-ATP6* variants, e.g., an overlap in some patients with non-syndromic neurological manifestation and in asymptomatic individuals. Furthermore, patients across all subtypes harbouring an *MT-ATP6* variant tend to have recurrent acute metabolic and physical decompensation during illness [3]. 

Here, we present a novel variant in the *MT-ATP6* gene in a 31- year old female with the clinical leading symptoms of proximal tetraparesis, insulin-dependent diabetes mellitus, recurrent lactic acidosis, and ketoacidosis during recurrent infections. As in most cases with *MT-ATP6* variants, muscle biopsy and respiratory chain activities were normal. Single-exome sequencing revealed a homoplasmic variant in the *MT-ATP6* gene: m.9143T>C. Segregation studies were performed in different samples of the index patient and her mother, who did not show any evidence for a neuromuscular disorder. Interestingly, the m.9143T>C variant is reported on mitoMAP and GenBank (GQ119047) as a variant [11]. However, no clinical information was given and the mutation is not reported as pathogenic. 

The presented case extends the phenotypic spectrum of reported *MT-ATP6* mutations by recurrent infections and immunodeficiency as a possible key symptom. 

## 2. Materials and Methods 

### 2.1. Clinical Description

Here, we present a 31- year old female who developed insulin-dependent diabetes mellitus at the age of 26 years. From there on, she suffered from recurrent lactic- and ketoacidosis, recurrent infections, sometimes requiring ventilation as well as severe muscle and limb pain and rapid physical exhaustion. Her medical history included bronchial asthma as well as suspected polyglandular autoimmune syndrome with type I diabetes mellitus with insulin pump therapy and autoimmune thyroiditis. She had a pulmonary artery embolism, suspected immunodeficiency with T cell lymphopenia and hypogammaglobulinemia, intervertebral disc herniation (L5/S1 left) requiring surgery as well as a bilateral cataract operation in the 30th year of life.

### 2.2. Histopathology and Activities of Respiratory Chain Complexes

Cryostat sections were cut from transversely orientated muscle blocks from the vastus lateral muscle of the patient and subjected to standard histological and histochemical analysis including COX (cytochrome C oxidase), succinate dehydrogenase (SDH) and COX-SDH oxidative enzyme reactions. Respiratory chain complex activities were determined spectrophotometrically according to standard protocols [12]. 

### 2.3. Molecular Genetic Studies

Exome sequencing and mtDNA analysis using off-target reads was performed as previously described [13]. For this purpose, DNA was extracted from peripheral whole blood. In brief, exome enrichment was done using an Agilent SureSelect Human All Exon Kit V6 (Santa Clara, CA, USA) and libraries were sequenced on an Illumina (San Diego, CA, USA) NovaSeq 6000. The mtDNA reads were aligned to the revised Cambridge Reference Sequence (rCRS) with the Burrows–Wheel Aligner (BWA) 0.7.5a using the mem algorithm. Variant calling was carried out with GATK 3.8. Variant filtering included a filter for putative biallelic non-synonymous variants (missense-, frameshift-, nonsense-, stoploss- and aplice-variants) with a minor allele frequency (MAF < 0.01) as well as heterozygous variants (MAF < 0.001). The latter were ranked based on a phenotype filter. For this purpose, an Online Mendelian Inheritance in Men (OMIM) full-text search was conducted with the search term “mitochondrial” and the respective gene from the results were queried for variants. The phenotype-based search could not identify (likely) pathogenic variants in genes associated with mitochondrial disorders in OMIM.

### 2.4. Determination of mtDNA Heteroplasmy Levels (RFLP Analysis)

For restriction fragment length polymorphism (RFLP) analysis, total DNA was extracted from different samples using the peqGOLD tissue DNA Mini Kit (Peqlab Biotechnologie GmbH, Erlangen, Germany) according to the manufacturer’s instructions. The presence of this variant m.9143T>C was determined by restriction digestion of the 220 bp PCR amplified product obtained using a forward 5′ ACC ATT AAC CTT CCC TCT ACA C 3′ and a reverse 5′ GAG GTC ATT AGG AGG GCT GAG A 3′ primer with TseI (New England Biolabs GmbH (NEB), Frankfurt am Main, Germany). The digested products were separated on agarose gels. The amplified fragments containing the mutation yielded two fragments of 67 and 153 bp, while the amplified wild-type fragment remained undigested (due to the loss of the restriction site). Triplet-analysis of mtDNA heteroplasmy (signal intensity of the bands) was carried out with ImageJ software.

### 2.5. Ethical Statement

The index patient and the mother gave written informed consent for study inclusion prior to analysis. The study was conducted in accordance with the Declaration of Helsinki and was approved by the Ethics Committee of the University Ulm (Project identification code 20/10) and of the Technical University in Munich.

## 3. Results

### 3.1. Clinical Findings

Clinical examination revealed mild proximal tetraparesis (upper extremities: MRC 4/5, lower extremities: MRC 3/5) with a positive Gowers’ phenomenon and residual sensorimotor L5 / S1 radiculopathy on the left leg with mild paresis of the foot-lifting (MRC 4/5) and lowering (MRC 4/5). No ptosis or ophthalmoplegia were found. Achilles tendon reflex could not be triggered on the left, the remaining deep tendon reflexes were normal and symmetrical and pathological reflexes could not be provoked. Muscle MRI (thigh muscles) showed normal findings. CK (creatine-kinase) was found to be normal (33 U/l; norm < 145U/l) even during metabolic decompensation and infections.

Neuropsychological examination revealed substantial cognitive impairment with reduced cognitive processing speed and dysfunction of attention, memory and word-finding. There were indications of relevant depressive symptoms. Conception, spontaneous speech, and social behaviour were inconspicuous. 

Neurography of the medial nerve, visually evoked potentials, and somatosensory evoked potentials of the medial and tibial nerve revealed normal findings on both sides. Electromyography detected a slightly increased number of turns at standard amplitude potentials, which was mostly compatible with a primary myopathic process. Transthoracic echocardiography was inconspicuous.

The mother did not report any symptoms of muscle weakness and did not show any evidence for a neuromuscular disorder during clinical examination nor any recurrent deterioration. Family history was inconspicuous for neuromuscular disorders and metabolic diseases. The patient has one brother who declined neurological examination and genetic testing.

### 3.2. Histopathology 

Muscle biopsy of the vastus lateral muscle showed no myopathic or neurogenic changes. There was no evidence of COX-deficient or ragged-red-fibres and only partially evidence for subsarcolemmal accentuation.

Biochemical analysis of the muscle from the index patient showed normal activities of respiratory chain complexes I, II/III, and IV as well as normal citrate synthase activity. 

### 3.3. Molecular Genetic Studies and Determination of mtDNA Heteroplasmy Levels 

Exome sequencing revealed a variant in the *MT-ATP6* gene: m.9143T>C (variant coverage: 22× and average coverage mtDNA: 52×) in a homoplasmic state which has not been published as pathogenic before. The amino acid position is highly conserved across species reflected by a PhastCons score of 0.978 and a PhyloP score of 3.94. The variant is predicted to disturb the C-terminal helical trans-membrane domain. The highest level of heteroplasmy in the index patient was found in muscle (99%) with lower levels present in urinary epithelial sediment (99%), buccal epithelial cells (96%), hair shafts (96%) and blood (95%) as determined by RFLP (Figure S1). Levels of mtDNA heteroplasmy detected in the patient’s mother are shown in Table 1.

## 4. Discussion

A disease causing variant in the *MT-ATP6* gene was first described in 1992 [4]. Since then, several cohort studies expanded the phenotypic spectrum, ranging from asymptomatic carriers to fatal early onset and multisystemic diseases [2,3]. Several “variants of unknown significance” (VUS) have been reported in the *MT-ATP6* gene. Due to the absence of clinically available CV activity testing, it remains challenging to fully assess if these variants contribute to clinical disease manifestations. 

Interestingly, the reported m.9143T>C variant is listed on mitoMAP and GenBank (GQ119047) as a variant in the Philippine population [11]. However, no clinical information was given and the mutation is not reported as disease causing. 

It is already known, that histochemical analysis of muscle biopsy and respiratory chain analysis are usually normal in patients with pathogenic *MT-ATP6* mutations, adding to the diagnostic challenges in the respective patients [1,3]. Though, definite pathogenic *MT-ATP6* variants are reported, in which standard biochemical findings can be subtle or inconsistent [1]. 

Generally, pathogenicity for mtDNA variants is proven by identifying the mtDNA variant to be present in symptomatic patients in a heteroplasmic state, rather than homoplasmy, and mtDNA variant heteroplasmy level in the affected patient being higher than in asymptomatic relatives [1]. Interestingly, it has already been shown that these approaches may be particularly problematic in the specific case of *MT-ATP6* variants [1]. In a recently published cohort study [3], the majority of patients presented with variable non-syndromic features including ataxia, neuropathy and learning disability. Maternal inheritance was confirmed in 39 families and demonstrated that tissue segregation patterns and phenotypic threshold are variant dependent [3]. The difference of the mutant heteroplasmy levels between different tissues (blood, urinary epithelial cells and buccal mucosal cells) was typically <10% in the majority of *MT-ATP6* variants reported in this study [3], which is in accordance to the abovementioned study and our results. In another cohort study, heteroplasmy levels were high in both clinically affected (mean 95%) and unaffected (mean 73%) individuals [2]. These findings were again consistent with our results, where the index patient and mother showed > 90% level of heteroplasmy in all tissues tested. However, it is already known, that the level of heteroplasmy did not clearly correlate with disease severity in several mitochondrial diseases [2,14]. In *MT-ATP6* variants, homoplasmic variants were even found in asymptomatic probands [2]. Further, due to the rapid heteroplasmy shifts that may occur in the level of *MT-ATP6* mutation load, pathogenic variants may appear to be homoplasmic and carrier patients may express symptoms which can be rather subtle [1]. As a result, apparently unaffected relatives may have *MT-ATP6* variant heteroplasmy levels that are high as that seen in clinically affected members of the same family [1]. Moreover, mutation loads of m.8993T>G [15,16,17], m.8993T>C and m.9185T>C overlap in some patients with non-syndromic neurological manifestation and in asymptomatic individuals [3]. This is absolutely concordant with our findings, where mother and index patient showed >90% level of heteroplasmy in all tested specimen. 

Acute metabolic and physical decompensation during intercurrent illness was documented in 27 patients harbouring a *MT-ATP6* mutation [3] which was seen in the presented patient, too. Interestingly, episodic metabolic decompensation (21/23 versus 6/36, *p* < 0.001) were significantly more common in patients with LS compared to those without LS [3]. It is already known that immunity and mitochondria (e.g., mitochondrial metabolism) are interlinked with each other [18,19], e.g., mitochondria can regulate activation and transcription of immune cells, differentiation and survival of immune cells. Furthermore, there is emerging evidence that both, autophagy and mitophagy play definitive roles in the control of mitochondrial homeostasis and regulation of innate and inflammatory responses [20]. Appropriately it was reported that in a cohort of 221 paediatric patients with mitochondrial disease, the global mortality rate was 14%, with sepsis (55%) and pneumonia (29%) being the two most common causes of death [21]. Furthermore, there are some animal models, underlying the connection of immunological dysfunction and mitochondrial diseases [22,23]. The presented index patient had a history of recurrent lactic acidosis and ketoacidosis; an immunodeficiency with T cell lymphopenia and hypogammaglobulinemia was suspected. However, it remains indistinct, if recurrent illness may be a part of the *MT-ATP6* mutation associated disease spectrum.

## 5. Conclusions

The present study provided important insights in the phenotypic spectrum of disease-causing *MT-ATP6* variants. Especially against this background, mitochondria are known to play a key role in immunity and regulation of innate and inflammatory responses. It can be concluded that recurrent infections and immunodeficiency may be a possible key symptom in *MT-ATP6*-related variants. 

## 6. Limitations

Since the asymptomatic proband’s mother showed very similar, nearly homoplasmic values of heteroplasmy, the pathogenicity of this variant is not easy to ascertain. Further functional studies are needed to assign definitive pathogenicity.

## Figures and Tables

**Table 1:** Segregation study to determine the level of mtDNA heteroplasmy (%) of the novel *MT-ATP6* variant in the index patient and mother; values given as means (%) by triplet-analysis + SD.

Tissue	Index	Mother
Muscle	99 ± 1.1	
Urinary Epithelial Sediment	99 ± 1.5	96 ± 1.1
Buccal Epithelial Cells	96 ± 1.5	94 ± 1.6
Hair Shafts	96 ± 1.7	96 ± 3.1
Blood	95 ± 0.6	94 ± 0.7

## Supplementary Materials

The following are available online at https://www.mdpi.com/2079-9721/8/2/19/s1, **Figure S1:** Determination of mtDNA heteroplasmy levels (RFLP analysis) in the index and patient’s mother on agarose gels.
