# Supplementary material for: The m.9143T>C Variant: Recurrent Infections and Immunodeficiency as an Extension of the Phenotypic Spectrum in MT-ATP6 Mutations?
Source: Diseases. 2020 Jun 9;8(2):19. doi: 10.3390/diseases8020019 (PMC7348873; doi:10.3390/diseases8020019)
Supplement: Supplementary file 1 [file diseases-08-00019-s001.pdf]

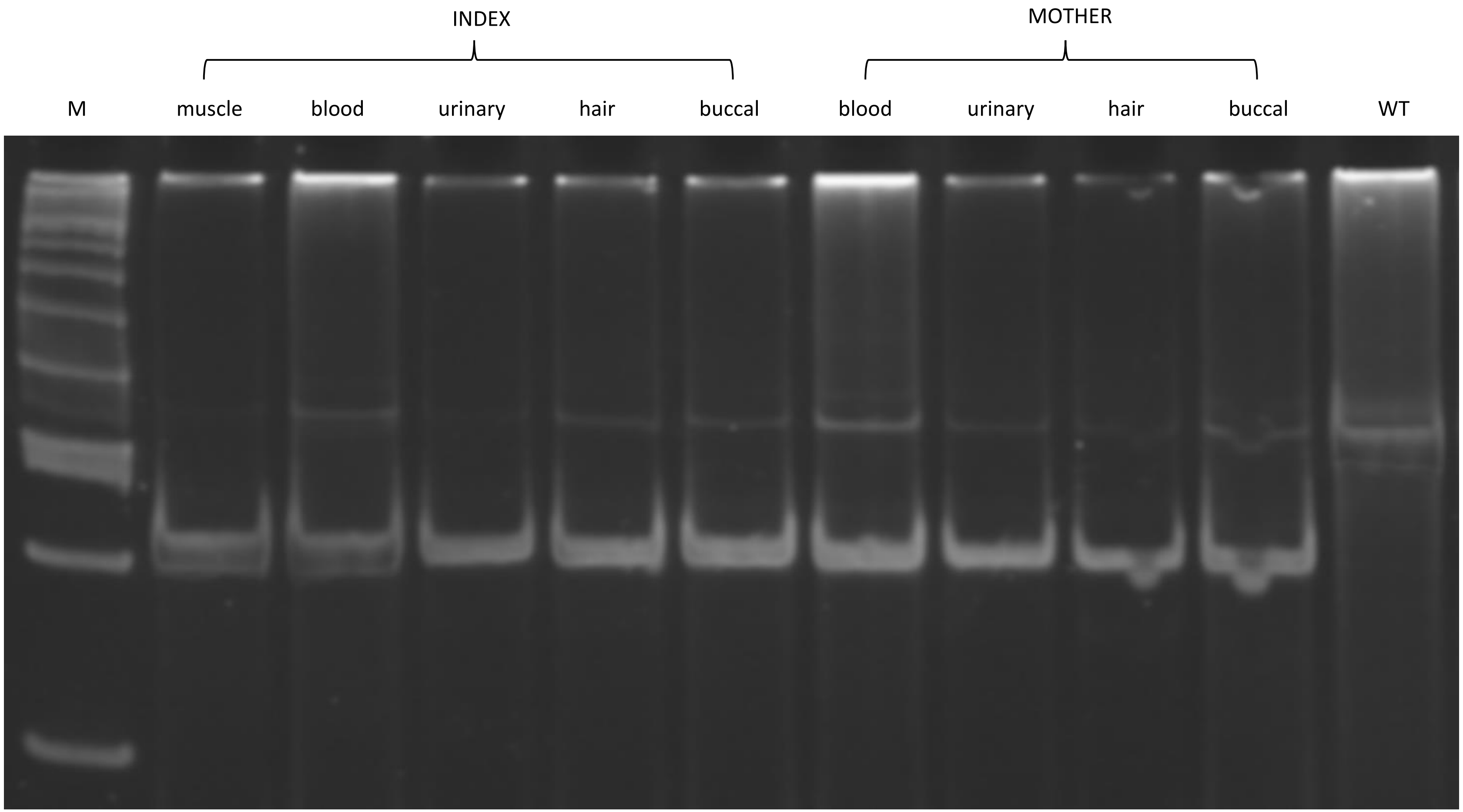

Figure S1: Determination of mtDNA heteroplasmy levels (RFLP analysis) in the index and patient's mother on agarose gels. The amplified fragments containing the mutation yielded two fragments of 67 and 153 bp. M = Marker, WT = wild- type.
